# 

**Published:** 1897-02-06

**Authors:** 


					THE HOSPITAL. ABSOLUTELY PURE, therefore BEST, Fbb. 6th, 1897,
CADBURY'S CADBURY'S
COCOA
at^^le!^-Z?^?StPUrit7atPreMnl NO ALKALIKS USED,
COOOA
-T    - - _ ?   ORMCH HOSPAJAL ?-
isoorr Western I hf lam art ~~ tjtjtpp >pivnpvirnv
m *u^3"#fULA5QOir W?STERN luFlOMA
No. 5417s*Vol. XXI.
oATTTRDAY,
Feb. 6th, 1897.
[Registered at the General Post Office as a Newspaper for Per Annum, 10s. 6<L, post free,
transmission in the United Kingdom and Abroad.] Monthly Parte, 6d.; post free, 8d.
PRICE TWOPENCE.
Per Ann am, 10s. 6<L, post free.
Monthly Parts, 6d.; post free, 8d.
Ditto per Annum, 8s. 6d., port free.

				

